# Self-reported sleep disturbances are associated with poorer cognitive performance in older adults with hypertension: a multi-parameter risk factor investigation

**DOI:** 10.1017/S1041610219001492

**Published:** 2020-07

**Authors:** Jordan N. Kohn, Emily Troyer, Robert N. Guay-Ross, Kathleen Wilson, Amanda Walker, Chad Spoon, Christopher Pruitt, Gary Lyasch, Meredith A. Pung, Milos Milic, Laura S. Redwine, Suzi Hong

**Affiliations:** 1 Department of Psychiatry, University of California, San Diego, USA; 2 Department of Family Medicine and Public Health, University of California, San Diego, USA; 3 Department of Medicine, University of California, San Diego, USA; 4 College of Nursing, University of South Florida, Florida, USA

**Keywords:** sleep, cognitive impairment, metabolic syndrome, physical mobility, inflammation, blood pressure, depressed mood, dementia

## Abstract

**Objectives:**

Given the evidence of multi-parameter risk factors in shaping cognitive outcomes in aging, including sleep, inflammation, cardiometabolism, and mood disorders, multidimensional investigations of their impact on cognition are warranted. We sought to determine the extent to which self-reported sleep disturbances, metabolic syndrome (MetS) factors, cellular inflammation, depressive symptomatology, and diminished physical mobility were associated with cognitive impairment and poorer cognitive performance.

**Design:**

This is a cross-sectional study.

**Setting:**

Participants with elevated, well-controlled blood pressure were recruited from the local community for a Tai Chi and healthy-aging intervention study.

**Participants:**

One hundred forty-five older adults (72.7 ± 7.9 years old; 66% female), 54 (37%) with evidence of cognitive impairment (CI) based on Montreal Cognitive Assessment (MoCA) score ≤ 24, underwent medical, psychological, and mood assessments.

**Measurements:**

CI and cognitive domain performance were assessed using the MoCA. Univariate correlations were computed to determine relationships between risk factors and cognitive outcomes. Bootstrapped logistic regression was used to determine significant predictors of CI risk and linear regression to explore cognitive domains affected by risk factors.

**Results:**

The CI group were slower on the mobility task, satisfied more MetS criteria, and reported poorer sleep than normocognitive individuals (all *p* < 0.05). Multivariate logistic regression indicated that sleep disturbances, but no other risk factors, predicted increased risk of evidence of CI (OR = 2.00, 95% CI: 1.26–4.87, 99% CI: 1.08–7.48). Further examination of MoCA cognitive subdomains revealed that sleep disturbances predicted poorer executive function (β = −0.26, 95% CI: −0.51 to −0.06, 99% CI: −0.61 to −0.02), with lesser effects on visuospatial performance (β = −0.20, 95% CI: −0.35 to −0.02, 99% CI: −0.39 to 0.03), and memory (β = −0.29, 95% CI: −0.66 to −0.01, 99% CI: −0.76 to 0.08).

**Conclusions:**

Our results indicate that the deleterious impact of self-reported sleep disturbances on cognitive performance was prominent over other risk factors and illustrate the importance of clinician evaluation of sleep in patients with or at risk of diminished cognitive performance. Future, longitudinal studies implementing a comprehensive neuropsychological battery and objective sleep measurement are warranted to further explore these associations.

## Introduction

Declines in cognitive function that accompany aging are an urgent public health concern due to increases in life expectancy. Cognitive decline beyond what would be expected for a person’s age and education level is referred to as mild cognitive impairment (MCI), the incidence of which is estimated to be between 12% and 18% in individuals 60 years of age or older (Petersen, 2016). Progression of MCI to neurocognitive disorders (NCD), such as Alzheimer’s disease (AD), occurs in 8% to 15% of cases annually (Petersen, 2016) and the incidence of NCD diagnoses is expected to triple over the next few decades and projected to reach 135 million individuals by the year 2050 (Prince *et al*., 2016) Different domains of cognitive function, including visuospatial, executive function, attention, language, memory, and orientation can be differentially affected in CI and NCD (Hugo and Ganguli, 2014). Several risk factors for cognitive decline among older adults have been identified, including sleep disturbances, hypertension (HTN), MetS, chronic low-grade inflammation, decreased mobility, and neuropsychiatric symptoms such as depression (Assuncao *et al*., 2018; Barnes and Yaffe, 2011; Cooper *et al*., 2015; Newcombe *et al*., 2018; Yaffe *et al*., 2014). As many of these risk factors interact and are associated with aging, comorbidity is common, which poses a central challenge in assessing CI and NCD risk by disentangling the independent risk that one factor exerts on cognitive performance over another. Further understanding of the complex relationships between these factors and age-related cognitive decline are therefore, needed and remains a critical step toward reducing the growing burden of NCD.

Cardiometabolic dysfunction is a primary risk factor for CI in aging. HTN affects over three-quarters of adults over 65 years of age (Benjamin *et al*., 2018). HTN can contribute to NCDs via disruption of cerebral microvasculature and white matter integrity, thus leading to alterations in the subcortical networks that subserve key cognitive domains, such as executive function, attention, learning, and memory. More recently, HTN was shown to promote amyloid-β (Aβ) accumulation and tau hyperphosphorylation in the brain, both of which are hallmarks of AD pathology (Iadecola and Gottesman, 2019). MetS, which is a common comorbid condition of HTN, characterized by obesity, hyperglycemia, and dyslipidemia, is also a risk factor for cognitive decline and is far more prevalent in older (54.9%) versus younger (34.3%) US adults (Shin *et al*., 2018). MetS could confer risk for NCDs by exacerbating cerebrovascular dysfunction (e.g. insulin resistance), either through additive or interacting processes with HTN (Li *et al*., 2017; Tyndall *et al*., 2016). Given that MetS is a heterogenous group of factors, the individual components of which may exacerbate cognitive decline, the mechanisms by which MetS contributes to NCDs are multifaceted (Assuncao *et al*., 2018; Stoeckel *et al*., 2016).

Sleep disturbances are increasingly recognized as a risk factor for poor cognitive functioning and are highly prevalent in the aging US population, with over half of individuals aged 65 years or older reporting at least one sleep-related complaint (Foley *et al*., 1995). Furthermore, changes in sleep duration and architecture occur throughout the life span, and accelerate with increasing age, including decreased total sleep duration, efficiency, slow wave sleep (SWS), and rapid eye movement (REM) sleep, contrasted by increased time spent in Stage 1 and Stage 2 sleep. Sleep is also more fragmented in older adults who are more likely to experience both insomnia and nighttime awakenings (Kamel and Gammack, 2006), as are sleep-disordered breathing (SDB) conditions such as obstructive sleep apnea (OSA). A recent meta-analysis demonstrated that all-cause self-reported sleep disturbances were associated with increased incidence of all-cause NCDs, including AD and vascular dementia (Shi *et al*., 2018). Mechanisms by which sleep dysregulation could contribute to NCD are likely multidimensional and include increased neuronal apoptosis (Naidoo *et al*., 2008), Aβ deposition (Spira *et al*., 2013), cerebral hypoperfusion and glucose hypometabolism (Daulatzai, 2017), and systemic inflammation (Irwin, 2019). Conversely, NCDs could predispose individuals to developing sleep disturbances if neural networks related to sleep are affected by NCD neuropathology (Yaffe *et al*., 2014). Importantly, some older age-related alterations in sleep may lead to sleep medication use when not warranted, which has been linked to dementia (Cheng *et al*., 2017).

Aging is also a state of low-grade chronic inflammation (i.e. inflammaging) (Müller *et al*., 2019), which is associated with the aforementioned risk factors for cognitive decline such as cardiometabolic dysfunction and sleep disturbances (Irwin, 2019; Yaffe *et al*., 2004). Inflammation has been implicated in the pathogenesis of NCDs and AD (Swardfager *et al*., 2010). Given the evidence of multiparameter risk factors for cognitive outcomes, multidimensional investigations of how sleep, vascular, metabolic, immunologic, and behavioral factors impact various domains of cognition in older adults are warranted. While sleep disturbances, HTN, MetS, chronic low-grade inflammation, decreased mobility, and depressive symptoms have been identified as risk factors of MCI and NCD, the extent to which these often co-morbid conditions are related to global CI and various domains of cognitive performance in aging adults is less clear.

The current study aimed to investigate these multifactorial associations in aging adults with HTN by examining associations between risk factors (i.e. sleep disturbances, anti-HTN medications, MetS, monocyte inflammatory responses, decreased physical mobility, and depressive symptoms) and cognitive performance. We hypothesized that increased risk factor incidence (e.g. higher incidence of sleep disturbance) will be associated with poorer global cognitive performance (e.g. MoCA-derived cognitive impairment risk) and decreased cognitive scores within visuospatial, executive function, attention, and memory domains.

## Methods

### Participants

All participants gave written informed consent to the protocol, approved by the University of California, San Diego (UCSD) Institutional Review Board, and demonstrated sufficient understanding of the study via the UCSD Brief Assessment of Capacity to Consent (Jeste *et al*., 2007). One hundred forty-five hypertensive (130 ≤ SBP ≤ 170 mmHg), regardless of antihypertensive medication use, non-smoking men and women between 60 and 90 years were recruited from the local community for a parent Tai Chi and healthy-aging intervention study. Initial screening via telephone interviews, followed by face-to-face confirmation, established the absence of these exclusion criteria: inability to perform light to moderate exercise, English-language illiteracy, regular planned moderate exercise or meditation practice (≥ 2 x week and ≥ 30 min per episode), recent stroke or cerebral neurological impairment, antipsychotic medication use, current major depressive disorder, psychosis or substance-use disorder, inflammatory disorders, or health-related factors affecting immune function (e.g. vaccinations <10 day, active infections/illness, immunomodulatory medication). A power analysis determined that a sample size of approximately 150 would be required to detect small-to-medium (*r* = 0.23) effects at 80% power with alpha = 0.05.

### Clinical evaluations

Average basal systolic blood pressure (SBP) and diastolic blood pressure (DBP) were calculated from three consecutive seated measurements on the left arm at 5 min intervals following 15 min seated rest in a dimmed, climate-controlled room using an automated oscillometric sphygmomanometer (Colin Press-Mate, model BP-8800, Komaki City, JP). Demographic variables and medical history were recorded via standardized interview. All medications were visually inspected and recorded from the prescribing label. Anti-hypertensive medications included beta-blockers, calcium-channel blockers, angiotensin-converting enzyme inhibitors, angiotensin-receptor blockers, and diuretics. Two participants did not provide medication data. Anthropometrics, including height, weight, and waist circumference (WC), were collected via tape and balance beam scale. Body mass index (BMI) was calculated as weight in kg/(height in m)^2^. Timed Up and Go (TUG) was used to assess physical mobility and is associated with cognitive performance in older adults(Ibrahim *et al*., 2017). TUG was quantified by the time (seconds) required to stand from a seated position, walk 3 meters, turn, and return to the chair, whereby longer time indicates poorer mobility. Blood was obtained between 1000–1200 for all participants after ≥ 12 h abstinence from caffeine into anti-coagulant vacutainers (BD, Franklin Lakes, NJ). Seven participants (4.8%) did not provide blood due to difficulties with i.v. collection. Lipids, glucose levels, glycosylated hemoglobin A1c (%HbA1c), and complete blood counts (to rule out infection and high hemoconcentration) were assessed by a commercial laboratory (LabCorp, Burlington, NC).

MetS was identified by the presence of ≥ 3 of the following five traits: (1) WC >94 cm in men or >80 cm in women, (2) plasma glucose ≥ 100 mg/dL, (3) systolic/diastolic BP ≥ 130/85 mmHg, (4) triglycerides ≥ 150 mg/dL, and (5) high-density lipoprotein cholesterol <40 mg/dL in men or <50 mg/dL in women. Plasma glucose values were non-fasted, given the timing of blood collection (i.e. mid-morning), but levels were examined against %HbA1c. While fasted glucose is typically used in MetS assessment, HbA1c ≥ 5.7% performs similarly well to fasted glucose ≥ 100 mg/dL (Ong *et al*., 2010). In this sample, blood glucose levels strongly correlated with %HbA1c (*r*_136_ = 0.81, *t* = 15.9, *p* < 0.001); a value of 100 mg/dL glucose corresponded to 5.78 %HbA1c.

Given that hypertension was an inclusionary requirement, all participants had ≥ 1 MetS trait. Depressive symptoms were assessed using the 21-item Beck Depression Inventory (BDI-II), which was selected over the Geriatric Depression Scale (GDS), specifically for its inclusion of somatic symptomatology commonly experienced in the elderly (Norris *et al*., 2004), and the relationship of somatic complaints to other risk factors such as poor sleep and cardiometabolic function. Sleep disturbances were evaluated using the eight-item short form Patient-Reported Outcomes Measurement Information System Sleep Disturbance (PROMIS-SD) scale. This scale measures self-reported perceptions of sleep quality, depth, and restoration within the past seven days, which includes perceived difficulties falling and staying asleep, as well as sleep satisfaction. Importantly, the PROMIS-SD was recently validated in a similar cohort of older adults (Full *et al*., 2019), and higher scores are significantly negatively correlated with actigraphy-based, objective measures of total sleep time (Hanish *et al*., 2017).

### Cognitive assessment

Participants were categorized as putatively “cognitively impaired” or “normocognitive (NC)” based on a 30-item MoCA score cutoff of 24/25. Although an MoCA cutoff score of 25/26 was initially proposed to indicate CI, subsequent studies indicate score dependence upon demographics and clinical comorbidities, and as such, there is no consensus on the optimal cutoff in the literature. Based on a recent meta-analysis indicating that a total score cutoff of 24/25 improved specificity and sensitivity for CI in older populations with ≥ 7 years of education (Davis *et al*., 2015), we opted for a 24/25 cutoff score. Domain-specific MoCA subscale scores reflecting six cognitive domains with convergent validity against standardized neuropsychological testing (Freitas *et al*., 2012) were computed for the following: visuospatial, executive function, attention, language, memory, and orientation.

### Intracellular monocyte TNF-α quantification

To assess regulatory processes of cellular inflammation, stimulated monocyte assays were performed using heparinized venous blood within 1 h of collection. Lipopolysaccharide (LPS; 200 pg/mL) (E.coli 0111:B4, #L4391, Sigma-Aldrich, St. Louis, MO) was added to 300 μL blood and incubated for 3.5 hrs at 37°C with 5% CO_2_ in 96-well plates, along with a non-LPS-treated sample (Dimitrov *et al*., 2013; Kohn *et al*., 2019). The proportion of CD14^+/dim^HLA-DR^+^ cells that were TNF-α^+^ was determined using FlowJo (v10, TreeStar, Ashland, OR). Beta-adrenergic receptor-mediated inflammation control (BARIC) was based on the inhibitory effect of isoproterenol (Iso), a β-AR agonist, on monocyte TNF-α production upon LPS stimulation. Monocyte β-AR-mediated TNF-α inhibition by Iso (i.e. BARIC) was calculated as percent change in %TNF-α^+^ monocytes between LPS-treated and LPS + Iso-treated samples. Samples were run in duplicate for 62 (58%) participants in order to establish reliability of the assay (intraassay coefficient of variability = 12.4%). More negative BARIC values indicate greater βAR responsivity (i.e. better β-AR-mediated inflammation regulation), and values near or equal to zero indicate little to no effect of Iso. One participant had a BARIC score >25% (indicating technical error), which was omitted by pairwise deletion.

### Statistical analysis

Descriptive statistics were computed in initial analyses. Continuous variables were compared between groups using the independent samples *t*-test (parametric) and Kruskal-Wallis*H*-test (non-parametric) for Gaussian and non-Gaussian distributions, respectively. Categorial variables were compared between groups using Pearson’s X^2^ test. Distributions of all variables were visually inspected for outliers. Univariate correlations between risk factors and cognitive outcomes were computed using Spearman’s *r* (*r*_*S*_) in order to examine associations of separate risk factors with cognitive outcomes. Statistical significance threshold for univariate correlations was adjusted for multiple comparisons (false discovery rate; FDR) using the Benjamini–Hochberg correction (66 total comparisons). Binomial logistic regression was implemented to determine odds ratios (ORs) for each risk factor in predicting cognition group membership. Non-parametric random-*x* resampling with 5,000 iterations was used to generate bias-corrected bootstrapped 95% and 99% confidence intervals (Fox and Weisberg, 2012), and significance was evaluated by bootstrapped 99% confidence interval. Goodness of fit was assessed using the Hosmer–Lemeshow test, and pseudo-R^2^ was calculated using Nagelkerke’s statistic.

Relationships among risk factors and cognitive domain scores were further assessed using multivariate linear regression. Bootstrapped beta estimates (5,000 iterations) established bias-corrected 95% and 99% confidence intervals, from which inferences were made regarding the null hypothesis that true associations did not exist between risk factors and domain-specific cognitive performance (Fox and Weisberg, 2012). Age, sex, native language, and number of antihypertensive drugs were included as covariates. BDI and PROMIS-SD scores were weighted for item nonresponse by multiplying raw scores by the inverse proportion of responded items. All model predictors were added in one step after being standardized (mean = 0, SD = 1). Statistical analyses were performed using R (v. 3.5.0). All tests were two-tailed; alpha level was 0.05. Residuals were tested for homoscedasticity using the Breusch–Pagan test, and normality assessed using the Wilks–Shapiro test. Studentized residuals and variance inflation factors were <3.0 for all predictors.

## Results

### Demographics and risk factors by cognitive subgroup

Table 1 shows the demographic characteristics, risk factors, and cognitive subdomain scores of the study participants grouped based on a MoCA score cutoff of 24/25. Participants (*N* = 54) in the CI group (MoCA ≤ 24) performed significantly worse on five of the six cognitive subdomains (all *p* < 0.001; except orientation *p* > 0.05) than participants (*N* = 91) in the NC group (MoCA ≥ 25). Age, sex, SBP and DBP, BDI-II score, cellular inflammation index (i.e. BARIC), and individual metabolic parameters did not differ between groups. However, the incidence of MetS risk factors was greater in the CI group (*H* = 5.95, *p* = 0.01). The number of concurrent antihypertensive medications taken was also significantly greater in the CI group (1.42 ± 0.16 medications) compared to the NC group (0.96 ± 0.10 medications; *H* = 5.32, *p* = 0.02), which was primarily driven by greater use of beta blockers (37% versus 20.9%, X^2^ = 4.62, *p* = 0.03). Statin drugs were also prevalent in the sample (36.6%) but did not differ between CI risk groups (X^2^ = 1.44, *p* > 0.05). TUG times were also significantly longer in the CI (10.3 ± 0.55 s) than in the NC group (9.1 ± 0.33 s; *H* = 5.12, p = 0.02), and PROMIS-SD scores were significantly higher in the CI group (23.1 ± 0.62 vs. 21.0 ± 0.33; *t* = 3.01, *p* = 0.003).

### Univariate associations of multi-dimensional risk factors with cognition

**Figure 1.**
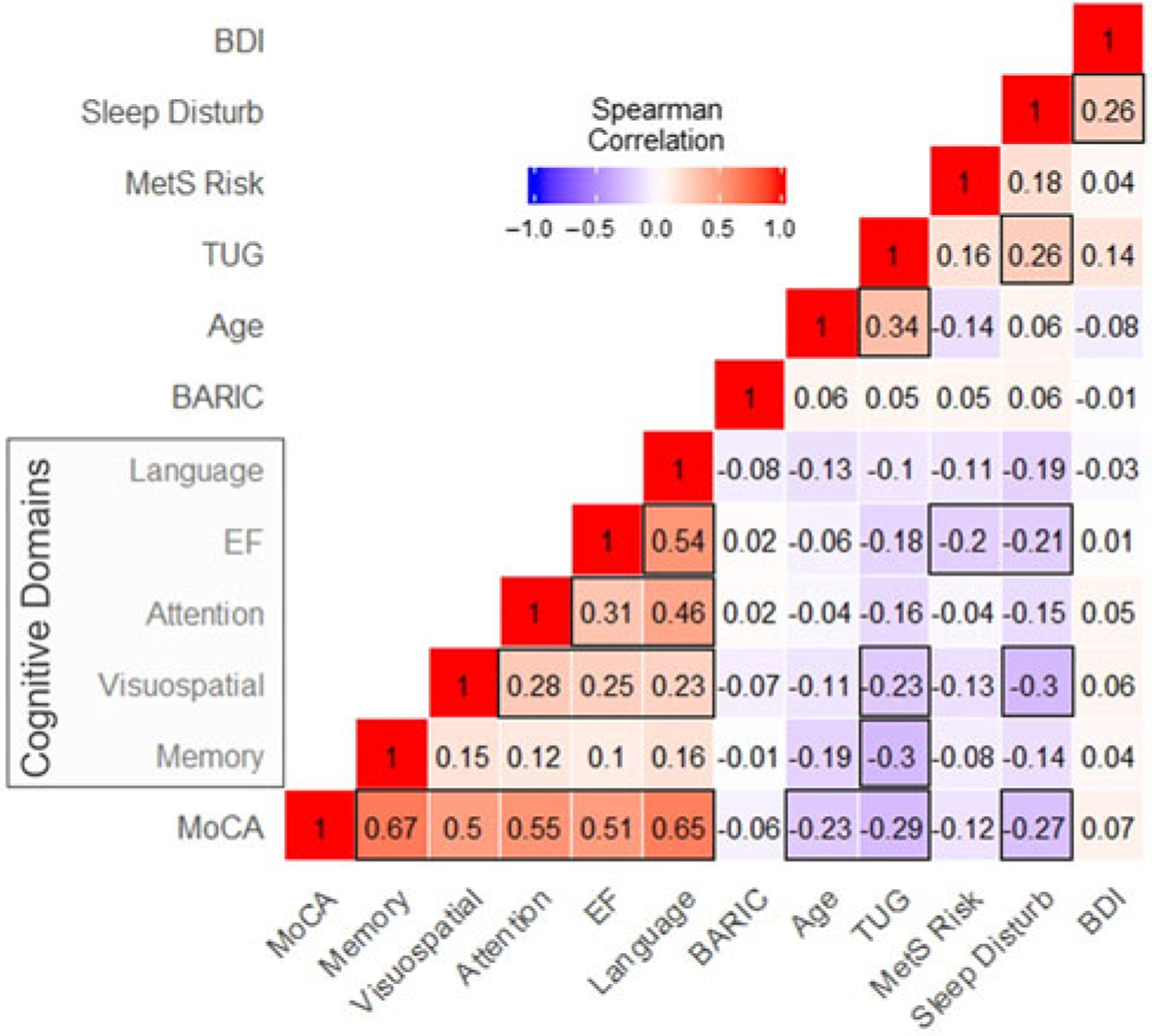
Univariate correlations.

Figure 1 shows univariate correlation coefficients between risk factors and MoCA total and subdomain scores. MoCA total scores were negatively correlated with age (*r*_*S*_ = −0.23), TUG time, indicating ambulatory functioning (*r*_*S*_ = −0.29), and PROMIS-SD, indicating sleep disturbance (*r*_*S*_ = −0.27) scores (all FDR-adjusted *p* < 0.05). PROMIS-SD scores were also negatively correlated with visuospatial (*ρ* = −0.30) and executive function (*r*_*S*_ = −0.21) subdomain scores (all adjusted *p* < 0.05). BDI scores and cellular inflammation were not associated with MoCA total or subdomain scores, though a higher incidence of MetS risk factors was associated with poorer executive function scores (*r*_*S*_ = −0.20). Higher PROMIS-SD scores were also associated with significantly longer TUG times (*r*_*S*_ = 0.26; *p* < 0.05) and higher BDI-II scores (*r*_*S*_ = 0.26; *p* < 0.05).

### Independent factors associated with increased risk of cognitive impairment

**Figure 2.**
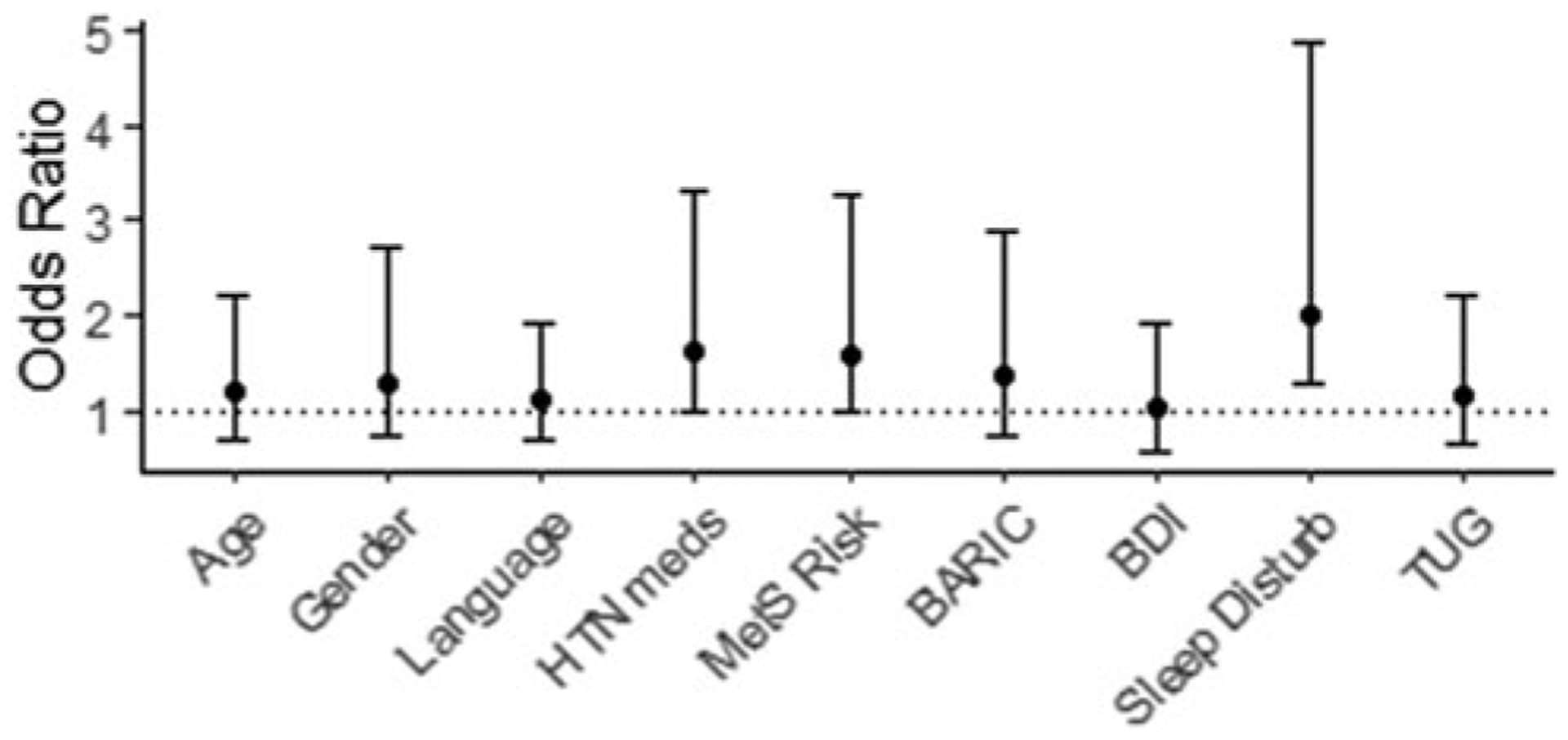
Effects of demographic and risk factors on risk of low MoCA score (≤ 24).

Logistic regression analysis was implemented to determine which risk factors predicted increased risk for evidence of cognitive impairment (MoCA ≤ 24). Figure 2 illustrates the odds ratios and bootstrapped confidence intervals for each predictor within a single regression model. Adjusted for age, sex, and native language, higher PROMIS-SD scores predicted a significantly increased risk of a low (≤ 24) MoCA total score [OR = 2.00, 95% CI: 1.26–4.87, 99% CI: 1.08–7.48] and explained 6.5% of the unique variance in risk (full model R^2^ = 38.2%). We did not find significant evidence of other risk factors conferring greater risk of a low MoCA score, including MetS risk factor incidence [OR = 1.59, 95% CI: 0.98–3.12, 99% CI: 0.78–4.86], and antihypertensive medication usage [OR = 1.60, 95% CI: 0.97–3.36, 99% CI: 0.81–4.06]. The remaining risk factors in the model, specifically TUG time, BDI score, and BARIC, showed no association with increased CI risk.

### Risk factors associated with cognitive subdomains

**Figure 3.**
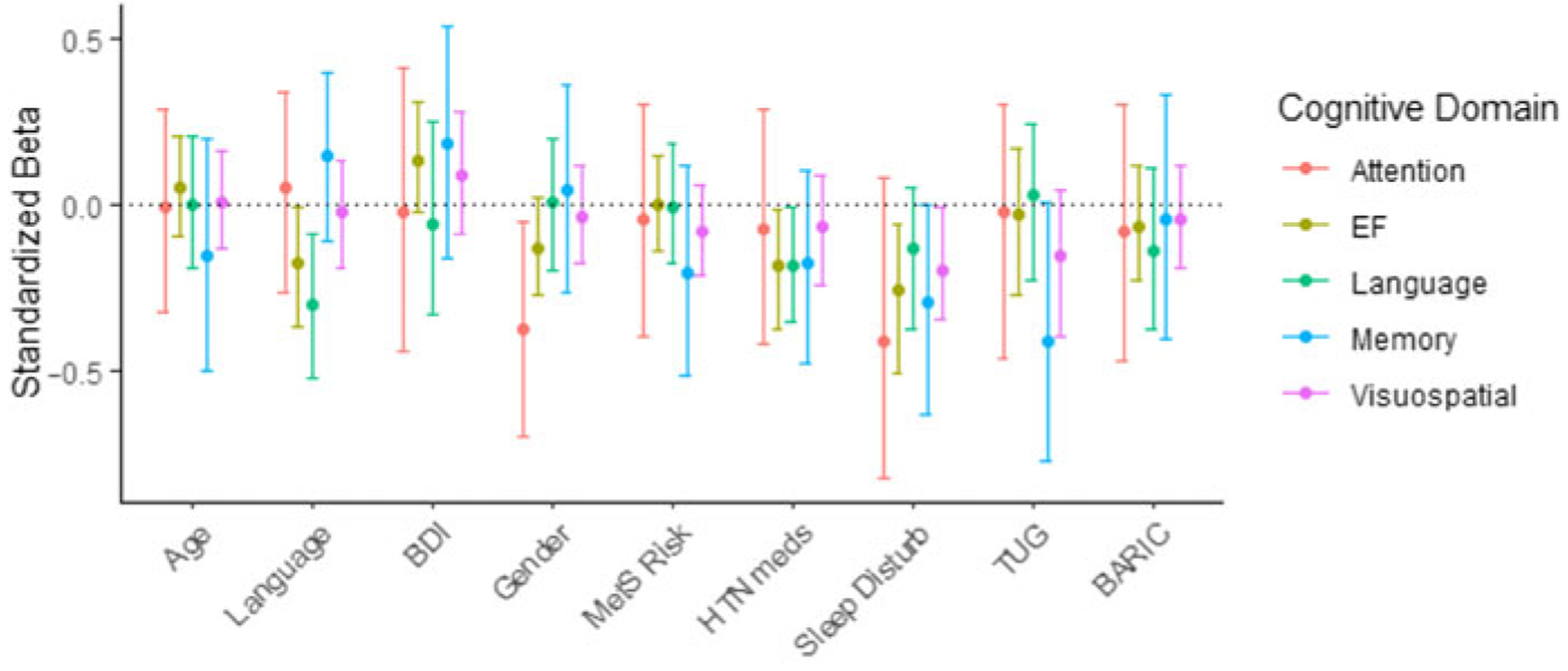
Effects of demographic and risk factors on MoCA cognitive subdomain scores.

In order to determine whether sleep disturbances or other risk factors were associated with poorer performance on specific cognitive domains, multiple linear regression analyses were implemented on each of the five MoCA subdomains that differed between CI and NC groups. Figure 3 shows the standardized effect sizes (i.e. beta coefficients) and bootstrapped confidence intervals for each predictor across the five regression models (one per subdomain). Adjusted for age, sex, and native language, higher PROMIS-SD scores were associated with poorer scores on executive function (β_std_ = −0.26, 95% CI: −0.51 to −0.06, 99% CI: −0.61 to −0.02), and somewhat lower visuospatial performance (β_std_ = −0.20, 95% CI: −0.35 to −0.02, 99% CI: −0.39 to 0.03) and memory (β_std_ = −0.29, 95% CI: −0.66 to −0.01, 99% CI: −0.76 to 0.08) domain scores of the MoCA. Antihypertensive medication usage was also associated with somewhat poorer performance on executive function (β_std_ = −0.19, 95% CI: −0.38 to −0.01, 99% CI: −0.44 to 0.05) and language (β_std_ = −0.19, 95% CI: −0.36 to −0.01, 99% CI: −0.41 to 0.06) subdomains.

## Discussion

We found that the deleterious impact of self-reported sleep disturbances on risk for CI and poorer cognitive performance was prominent beyond that of other more widely discussed clinical risk factors in our hypertensive, community-dwelling older individuals. Given the evidence of multiple and variable risk factors in determining cognitive outcomes in aging, our investigation examined the simultaneous impact of key clinical factors on CI risk across multiple domains, including physical mobility, cellular inflammation, cardiometabolic indices, depressive symptoms, and self-reported sleep disturbances as well as being independent of each other. Thus, our results provide the evidence that later-life sleep disturbances are a significant, independent risk factor for poorer cognitive performance, specifically within the domains of executive function, visuospatial and memory performance. Other potentially important factors that emerged include MetS and the number of different antihypertensive medications used by individuals in our sample.

Self-reported sleep disturbances can have numerous etiologies in older adults, such as SDB, nocturia, REM sleep behavior disorder (RBD), changes in sleep architecture, and longer sleep latency. The effects of sleep disturbances on cognitive decline and dementia have been well-documented, and robust evidence suggests a bidirectional relationship (Sindi *et al*., 2018; Yaffe *et al*., 2014) with multiple proposed underlying mechanisms. Decreases in SWS, the sleep phase during which metabolic waste is most efficiently cleared, may promote Aβ deposition (Ju *et al*., 2017) and neuronal apoptosis (Naidoo *et al*., 2008). Although not assessed in the current study, intermittent hypoxia due to SDB, as is the case with OSA, may cause neuronal impairment and CNS dysfunction (Feng *et al*., 2013), possibly through cerebral hypoperfusion, glucose hypometabolism (Daulatzai, 2017), and hyperactivation of brain microglial cells (Irwin, 2019). OSA is more prevalent in individuals with MetS and its associated factors (Lévy *et al*., 2015), such as obesity and HTN. Peripheral metabolic dysfunction (e.g. insulin resistance) is reflective of central metabolic stress (e.g. loss of CNS glucose transporter function), which can cause neuronal damage (Koren and Taveras, 2018). Sleep disruption may therefore increase risk of cognitive decline either independently, or by exacerbating other risk factors for cognitive decline. Conversely, NCDs could predispose individuals to developing sleep disturbances if neural networks related to sleep are affected by NCD neuropathology (Yaffe *et al*., 2014). For example, if neurons in the sleep-promoting ventrolateral preoptic area (VLPO) of the hypothalamus are affected, this could contribute to decreased sleep duration and increased fragmentation (Gaus *et al*., 2002), while loss of neuronal integrity in the suprachiasmatic nucleus (SCN) can be associated with abnormal circadian rhythm (Gubin and Weinert, 2015), and synucleopathies (e.g. Lewy body dementia) affecting brainstem nuclei are strongly associated with the development of RBD (Trotti, 2010), highlighting the bidirectional relationships between NCD and sleep.

We found significant associations between sleep disturbances and poorer executive function, visuospatial, and memory domain scores. Declines in executive function with aging are likely related to altered structure and function of frontal-striatal circuits, which are particularly sensitive to changes in white matter, and are preferentially observed in vascular dementia. This subclinical finding is notable given that participants in our study were not identified as sleep-disordered, and yet certain diagnosable sleep disorders are associated with deficits in the same cognitive domains as our finding (Bertrand *et al*., 2013). A recent meta-analysis indicates that low sleep efficiency, characterized by waking after sleep onset and less time in SWS, is also associated with poor executive function through multiple cortical mechanisms (Holanda Júnior and Almondes, 2016), whereas SDB affects executive function as well as memory and attention (Zimmerman and Aloia, 2012). In our sample, only five participants (3.5%) reported using sleeping medications, and none had ever received treatment for OSA, though we did not query histories of other diagnosed sleep disorders further in detail. Thus, it is plausible that some participants had not reported their condition or been subject to underdiagnoses. This raises the possibility that sleep disturbances were underreported or underdiagnosed in the elderly, which may extrapolate to sleep disorders as a frequently overlooked comorbidity in CI. Furthermore, no study participants had been formally diagnosed with CI; however, the associations between sleep disturbances and poorer performance in multiple cognitive domains, including executive function, visuospatial, and memory, suggests that screening for sleep disturbances in aging adults might be helpful in identifying individuals at risk for both non-amnestic and amnestic forms of CI.

Older adults with CI may have difficultly recalling and reporting more objective aspects of sleep, such as latency or duration. Interestingly, a recent longitudinal study in aging men found that sleep disturbances, but not daytime tiredness or sleep duration, were associated with a 58% increase in dementia after adjusting for known metabolic and inflammatory risk factors (e.g. C-reactive protein), and physical activity (Luojus *et al*., 2017). As discussed earlier, NCD-related neuropathology itself disrupts brain circuits and structures that are critical for normative sleep regulation, such as the ascending arousal system (Holth *et al*., 2017). Given the complexity and challenges of sleep disorders, the use of a rapid, easily administered self-reported sleep disturbance instrument may be indicated as a sensitive screening instrument to guide clinical decision-making in patients with or at-risk for cognitive impairment and dementia.

Although we identified CI-risk-group differences in physical mobility and the incidence of MetS criteria, multivariate analyses adjusted for demographic and clinical factors attenuated by these relationships. Physical mobility for our sample, which we assessed using the TUG task, was generally within age-matched, normative reference values (Bohannon, 2006). Recent work has suggested that physical *frailty* as a composite of slow gait, muscle weakness, unintended weight loss, low physical activity, and fatigue may serve as a mediator of sleep-cognition relationships in aging (Kaur *et al*., 2019). By comparison, our functional assessment, being unidimensional, may have required additional measurement domains in order to detect covariate-adjusted associations with the cognitive screen used in our analysis. Alternatively, our study sample, comprised of relatively high-functioning and ambulatory individuals, may have exerted a “ceiling effect” on such associations.

While the sample population was clinically hypertensive (SBP ≥ 130 mmHg), BPs were generally well-controlled. We found evidence for a relationship between the number of antihypertensive medications and CI risk, indicating a potential iatrogenic effect (e.g. polypharmacy) on cognitive performance, and/or that medication use serves as a proxy for hypertension severity and its cerebrovascular sequelae that influence cognition (Muela *et al*., 2017). Recent evidence (SPRINT MIND Investigators for the SPRINT Research Group *et al*., 2019) indicates that intensive pharmacologic treatment of hypertension to lower BP (to SBP ≤ 120 mmHg) in older adults reduces the risk of MCI over a 5-year follow-up compared to standard management (SBP ≤ 140 mmHg), which is in agreement with prior meta-analytic findings (Rouch *et al*., 2015). Notably in our sample, BPs did not differ between CI risk groups; however, these associations, plus evidence that hypotension may decrease cerebral perfusion, leading to hypoxia, neuronal death, and other sequelae (Sambati *et al*., 2014), highlights the importance of closely managed hypertension treatment and medication management to mitigate the negative effects of hypertension (or hypotension) on cognitive outcomes.

### Limitations

First, reliance on self-reported sleep disturbances and lack of objective sleep assessment, such as motor-activity (actigraphy) or sleep brain-wave (e.g. polysomnography), may have provided added validity of sleep quality. However, self-reported sleep disturbance is a valid and meaningful gauge of its clinical and cognitive impact and of high clinical utility with time- and cost-efficiency. Second, our cross-sectional experimental design would benefit from long-term follow-up so that prospective analysis could help disentangle causal relationships between sleep and cognitive impairment and dementia. This is important, as there remains a lack of consensus as to whether sleep disturbances in mid-life (e.g. 50–65 years old) are a stronger predictor of later-life (e.g. >65 years old) cognitive decline than later-life poor sleep. Another consideration is that the study population was predominantly women, which may limit the generalizability of our findings, particularly given sexual dimorphisms in age trajectories of inflammation, MetS, and other risk factors (Yang and Kozloski, 2011), as well as certain sleep disorders like RBD and sex-specific biases (e.g. selection, survivorship) in dementia-related research (Mayeda, 2019). Our findings of sleep disturbance association with a poorer cognitive performance are independent of sex, however. It should be noted that the lack of association between BDI-II scores and cognitive outcomes in our sample may be related to generally low levels of depressive symptoms (BDI-II total score ~7), as studies in clinically depressed and more severely symptomatic older adults have firmly established associations between depression and increased dementia risk (Byers and Yaffe, 2011). Lastly, the complex relationships between risk factors likely exert mediational and moderating (i.e. interaction) effects on cognitive performance, which is beyond the scope of this investigation and warrants a future study of a larger sample to enhance statistical power needed to detect such dynamics.

Despite these limitations, the present study benefited from several strengths. First, our assessments of cognitive performance (MoCA) with multi-parametric risk of sleep disturbances (PROMIS-SD), MetS, depressive symptoms (BDI-II), and physical mobility (TUG) are rapid to administer increasing utility, and readily translatable into the clinical setting. Indeed, geriatricians likely utilize the same tools already as part of routine care (e.g. glucose and lipid panels, anthropometrics), and can apply these findings to focus more acutely on sleep-cognition relationships, particularly using these screening instruments. Second, the study population was comprised of hypertensive, though otherwise, relatively healthy community-dwelling older adults, increasing the applicability to a large aging population, the majority of whom meet criteria for clinical hypertension (SBP > 130 mmHg). Thus, our findings may be particularly relevant to “normatively” aging adults without serious clinical conditions. Although, as a cross-sectional investigation we cannot assess causality, prospective studies do suggest that mitigating sleep disturbances may attenuate the risk of future (or further) cognitive decline. Thus, our results illustrate the importance of clinician evaluation of sleep in older adults with or at risk of diminished cognitive performance. Future, longitudinal studies implementing a comprehensive neuropsychological battery and objective sleep measurement are warranted to further explore these associations.

## Figures and Tables

**Table 1.** Participant characteristics

	VARIABLE	COGNITIVELY IMPAIRED (*N* = 54; 37%)	NORMOCOGNITIVE (N = 91; 63%)	STATISTIC (*T*, *H*, X^2^)
	Sex (%M/F)	20/80	32/68	2.24
	Age (years)	73.7 (8.3)	71.7 (7.7)	1.41
Metabolic	BMI (kg/m^2^)	29.2 (6.7)	29.5 (6.4)	0.24
Parameters	Waist circumference (cm)	102.5 (17.1)	100.6 (17.3)	0.65
	Glucose (mg/dL)	124.1 (41.9)	106.5 (28.7)	1.60
	HDL (mg/dL)	58.8 (19.5)	63.0 (18.1)	1.25
	Triglycerides (mg/dL)	147.7 (72.5)	130.7 (65.1)	1.39
	SBP (mmHg)	136.1 (19.0)	131.4 (17.6)	1.47
	DBP (mmHg)	68.8 (10.4)	68.8 (9.3)	0.00
Cognitive	MoCA total score (max = 30)	21.7 (3.0)	27.2 (1.6)	102.1**
Function	> Memory (max = 4)	1.79 (1.7)	3.81 (1.1)	43.4**
	> Executive function (max = 4)	2.81 (1.1)	3.70 (0.5)	35.6**
	> Visuospatial (max = 3)	1.83 (0.9)	2.59 (0.7)	28.0**
	> Language (max = 6)	4.28 (1.2)	5.52 (0.7)	38.5**
	> Attention/concentration (max = 8)	5.60 (2.2)	7.12 (1.2)	20.7**
	> Orientation (max = 6)	5.68 (0.8)	5.80 (0.5)	0.36
Risk Factors	TUG (sec)	10.3 (4.0)	9.1 (3.1)	5.12*
	Sleep disturbances (max = 40)	23.1 (4.5)	21.0 (3.1)	3.01**
	Depressive symptoms (BDI total)	7.62 (7.6)	6.76 (6.0)	0.11
	BARIC (%TNF suppression)	−40.7 (23.4)	−44.4 (20.5)	0.58
	Anti-HTN drugs (% taking/mean no.)	71.7% / 1.42	60.0% / 0.96	1.99 / 5.32*
	MetS risk-factor incidence (1–5)	3.24 (0.96)	2.77 (1.11)	5.95*

** *p* < 0.01,

* *p* < 0.05, based on independent *t*-test, Kruskal-Wallis *H*-statistic, or Pearson’s X^2^ test (categorical). Means and standard deviations shown. Cognitive impairment group defined as MoCA total score ≤ 24. Abbreviations: BMI = body mass index; HDL = high-density lipoprotein; SBP = systolic blood pressure; DBP = diastolic blood pressure; MoCA = Montreal Cognitive Assessment; TUG = Timed Up and Go task; Sleep disturbances = PROMIS Sleep Disturbance Scale score; BDI = Beck Depression Inventory-II; Anti-HTN drugs = antihypertensive medications (see Methods); MetS = metabolic syndrome.
